# Exploring changes in oral hygiene behaviour in patients with diabetes and periodontal disease: A feasibility study

**DOI:** 10.1111/idh.12365

**Published:** 2018-10-10

**Authors:** Katrin M. Jaedicke, Susan M. Bissett, Tracy Finch, Jared Thornton, Philip M. Preshaw

**Affiliations:** ^1^ Faculty of Health Sciences and Wellbeing University of Sunderland Sunderland UK; ^2^ Centre for Oral Health Research, School of Dental Sciences and Institute of Cellular Medicine Newcastle University Newcastle upon Tyne UK; ^3^ Department of Nursing, Midwifery & Health Northumbria University, Coach Lane Campus West Newcastle upon Tyne UK; ^4^ Newcastle Clinical Trials Unit Newcastle University Newcastle upon Tyne UK

**Keywords:** behaviour, diabetes mellitus, oral hygiene, qualitative research

## Abstract

**Objective:**

Exploring the feasibility to understand changes in oral hygiene behaviour using the Health Action Process Approach (HAPA) model applied to qualitative research interviews in patients with diabetes and periodontitis undergoing standard periodontitis treatment.

**Methods:**

Patients with type 1/2 diabetes and chronic periodontitis (n = 8) received standard non‐surgical periodontal treatment accompanied with personalized oral hygiene instructions by a dental hygienist. Clinical indices (% bleeding on probing (BOP), probing depth (PD), clinical attachment level (CAL), % of sites with PD ≥ 5 mm, periodontal epithelial surface area (PESA) and periodontal inflammatory surface area (PISA) were recorded pre‐ and post‐treatment. At 3 months post‐treatment, patients were interviewed using a topic guide relating to oral health. A behaviour change framework was constructed from elements of the HAPA model and used directly to map interview data to evaluate oral hygiene behaviour in these patients.

**Results:**

Data from this feasibility study suggest a clinical improvement in periodontal status, albeit only monitored for 3 months. Application of the HAPA model highlighted the behavioural change pathway that diabetes patients undertake before, during and after periodontal treatment. The data suggest that patients move through all elements of the motivation phase and all elements of the volition phase except for the recovery self‐efficacy element.

**Conclusion:**

The novel approach of applying the HAPA model to qualitative research data allowed for the collection of richer data compared to quantitative analysis only. Findings suggest that, in general, patients with periodontitis and diabetes successfully manage to incorporate new oral hygiene behaviours into their daily routine.

## 1 INTRODUCTION

Periodontitis is a chronic inflammatory disease affecting the tooth‐supporting structures, which requires lifelong management involving self‐care by the affected individuals, and professionally delivered care from dental professionals.^1^ Specifically, oral self‐care by patients has been noted to be of particular importance in achieving successful treatment outcomes.^2^, ^3^ To facilitate this, patients are given oral hygiene instructions (OHI) as part of their periodontal treatment plan and then typically receive 3‐monthly supportive periodontal care (maintenance care) recalls following initial periodontal therapy.^4^, ^5^ OHI aims to induce a behaviour change in the patients’ daily tooth cleaning regime, such as, for example, introducing the use of interdental brushes or the correct techniques when using a toothbrush.^5^

Research into psychological interventions for oral hygiene behaviour is based on a number of behavioural change theory models, such as Health Locus of Control, Social Learning Theory or Theory of Planned Behaviour.^6^, ^7^ Originally, behavioural change theory models were applied primarily to improve, for example, uptake of flu vaccinations, cancer screening attendance or to evaluate HIV risk behaviours.^8^ However, through continued development and refinement of these models, they have also found wide application in other areas, such as dentistry or diabetes care.^9^, ^10^ In primary care dental practice, patients receive OHI from their treating clinician. However, when applying psychological interventions, most research has been conducted with either psychologists or specifically trained dental personnel to provide the intervention. For example, research investigating motivational interviewing as an intervention to improve oral hygiene behaviour was conducted by a clinical psychologist or a trained counsellor.^11^, ^12^, ^13^ In addition to using specifically trained dental personnel, other studies have employed frequent visits and group classes with intense supervision of patient compliance to achieve a behavioural change in oral hygiene procedures.^14^, ^15^, ^16^, ^17^ It is therefore questionable how representative and feasible such approaches would be for everyday dental practice. Indeed, the most recent Cochrane review on psychological interventions to improve adherence to OHI highlighted that there is a lack of studies with practitioners other than trained specialists facilitating the psychological interventions.^6^

To evaluate how successful psychological interventions are in improving adherence to OHI, psychological constructs derived from behavioural change models are matched with clinical or questionnaire data outcomes. For example, psychological constructs such as locus of control, self‐efficacy, action‐planning or intention are correlated with tooth brushing, plaque index, flossing or questionnaire data in complex pathway analysis regression models.^18^, ^19^, ^20^ Specifically, the Health Action Process Approach (HAPA) model^21^ is one of the latest behavioural change theories applied to oral hygiene behaviour.^18^, ^20^, ^22^

The HAPA model is based on five principles instead of testable assumptions, which makes it distinct from other behavioural change models.^23^ The first principle, motivation and volition, is based on a division of the behaviour change process in first developing an intention and then making and acting on a decision. The second principle, two volitional phases, clarifies that the volition phase includes people at different stages of the behaviour change process, namely preintenders, intenders and actors. Principle three is based on postintentional planning, which includes people who are motivated to change but may lack the right skills to do so. Principle four, two kinds of mental simulation, divides the planning phase into two stages. The “when, where and how” of the intended action phase and the coping with barriers to the action phase. The fifth and final principle is phase‐specific efficacy. This perceived self‐efficacy runs throughout the whole behavioural change process, however, changes in nature from task self‐efficacy to maintenance and finally recovery self‐efficacy.^23^

Whilst the approach to correlate psychological constructs with clinical and questionnaire data results in clear quantitative outcome measures, it does not take patients’ emotions and feelings into account and does not identify where patients may be struggling with implementing the required behaviour change. Alternatively, using qualitative research interviews to explore behavioural changes in oral hygiene may provide an overall richer analysis and be especially relevant in a patient group that already has to manage other chronic condition such as diabetes or cardiovascular diseases.

Diabetes and periodontitis are both recognized as chronic inflammatory conditions, linked through immunological changes in inflammatory cytokine networks.^24^ This two‐way relationship makes patients with diabetes a particularly vulnerable group for developing periodontitis, having a threefold increased risk compared to individuals without diabetes.^25^ Patients with diabetes are having to make lifelong changes in their diet and exercise regimes, coping with frequent checks of blood glucose levels and numerous routine medical appointments.^10^ Hence, there is a reasonable expectancy that this patient group, in particular, may struggle with fitting in the additional task of caring for their oral hygiene when diagnosed with periodontitis.

The aim of this study therefore was to investigate whether it is feasible to understand a behavioural change in oral hygiene using the HAPA model through qualitative interview analysis in patients with diabetes and periodontal disease who have received standard care only and not a specialized psychological intervention.

## 2 METHODS

### 2.1 Study population

This was a longitudinal observational feasibility study in patients with diabetes and chronic periodontitis. Eight adult patients with type 1 or type 2 diabetes and chronic periodontitis were recruited from periodontology referral clinics at Newcastle Dental Hospital, Newcastle upon Tyne, UK. Patients were recruited to the study if they were adult males or females of age 18‐65 years, had a confirmed diagnosis of diabetes (diabetes is defined as HbA1c > 6.5%, in accordance with current diagnostic guidelines^26^), a minimum of 20 natural teeth (excluding third molars), were willing and able to comply with study procedures and had chronic periodontitis. Chronic periodontitis was defined as the presence of interproximal probing depth (PD) of ≥5 mm at ≥1 site at ≥1 tooth, together with per cent bleeding on probing (%BOP) scores ≥10%. Exclusion criteria included the presence of infectious systemic or oral diseases, bleeding disorders, pregnant or nursing mothers, or patients who had received a professional dental prophylaxis within the last 4 weeks. General characteristics of the study population are presented in Table 1.

**Table 1:** General and clinical characteristics of the study population pre‐ and post‐treatment

	Pre‐treatment	Post‐treatment
n [male/female]	5/3	
Age [y]	61.5 ± 39	–
BMI [kg/m^2^]	27.8 ± 11.2	
Ethnicity [n]		
Caucasian	6	
Black	2	–
Asian	–	
Smoking status [n]		
Current	–	
Ex	1	–
Non	7	
BOP [%]	30 ± 93	19 ± 66
Sites with probing depths ≥5 mm [%]	37.6 ± 66.8	25.5 ± 51.2
CAL [mm]	4.9 ± 2.2	3.8 ± 2.6
PD [mm]	3.7 ± 2.8	3.4 ± 2.4
PESA [mm^2^]	2202 ± 1966	1566 ± 2340
PISA [mm^2^]	623 ± 2921	447 ± 1719

Data shown are median ± interquartile range.

BMI, body mass index; BOP, bleeding on probing; CAL, clinical attachment level; PD, probing depth; PESA, periodontal epithelial surface area; PISA, periodontal inflammatory surface area.

### 2.2 Periodontal treatment and indices

Full mouth periodontal indices (% bleeding on probing (BOP), probing depth (PD), clinical attachment level (CAL), % of sites with PD≥5 mm, periodontal epithelial surface area (PESA) and periodontal inflammatory surface area (PISA) were recorded at 6 sites per tooth using a UNC‐15 periodontal probe pre‐treatment and at 3 months after treatment. Patients received standard non‐surgical periodontal treatment that was provided by a dental hygienist using a full mouth debridement approach (ie, full mouth treatment within as short a time period as possible, typically 2‐7 days) with manual and ultrasonic instruments and local anaesthetic as indicated clinically. Patients were treated in one or two visits, depending on their preferences and clinical needs. Patients were seen by the same dental hygienist (not one of the researchers involved in the study) working at the periodontology referral clinic throughout the whole research.

### 2.3 Oral hygiene instruction

As part of their periodontal treatment, each patient received personalized OHI from the dental hygienist to motivate them and encourage a high standard of oral hygiene. This advice was based on guidance from the National Institute for Health and Care Excellence (NICE).^27^ In brief, patients were given an overview of periodontal risk‐factors (eg, diabetes, smoking, family history, specific medications) and were shown pictures to explain the appearance of periodontal health, gingivitis and periodontitis. Patients were asked to use their own toothbrush in front of the dental hygienist holding a mirror and instruction to improve their technique was provided whilst doing so. The OHI was tailored to each patient's clinical needs taking into consideration their demonstrated abilities to use oral hygiene products and the extent and severity of their periodontitis. Patients were provided with interdental brushes with specific instruction into their use (eg, the correct sizing of interdental brushes according to the spacing between teeth).

### 2.4 Qualitative research interviews and HAPA model application

Research interviews based on a topic guide relating to oral health were conducted at the 3 months review visit prior to the dental hygienist recording periodontal indices, and were audio recorded. The topic guide included subjects such as the patients’ tooth brushing and cleaning routine, reasons why the patients attended the dental hospital, patients’ awareness of their periodontitis prior to the dental hospital visit, patients’ diabetes management and how they have incorporated the new OHI. Each interview lasted approximately 20‐30 minutes. Interviews were transcribed verbatim and analysis was managed using software (NVivo, QSR International).

**Figure 1:**
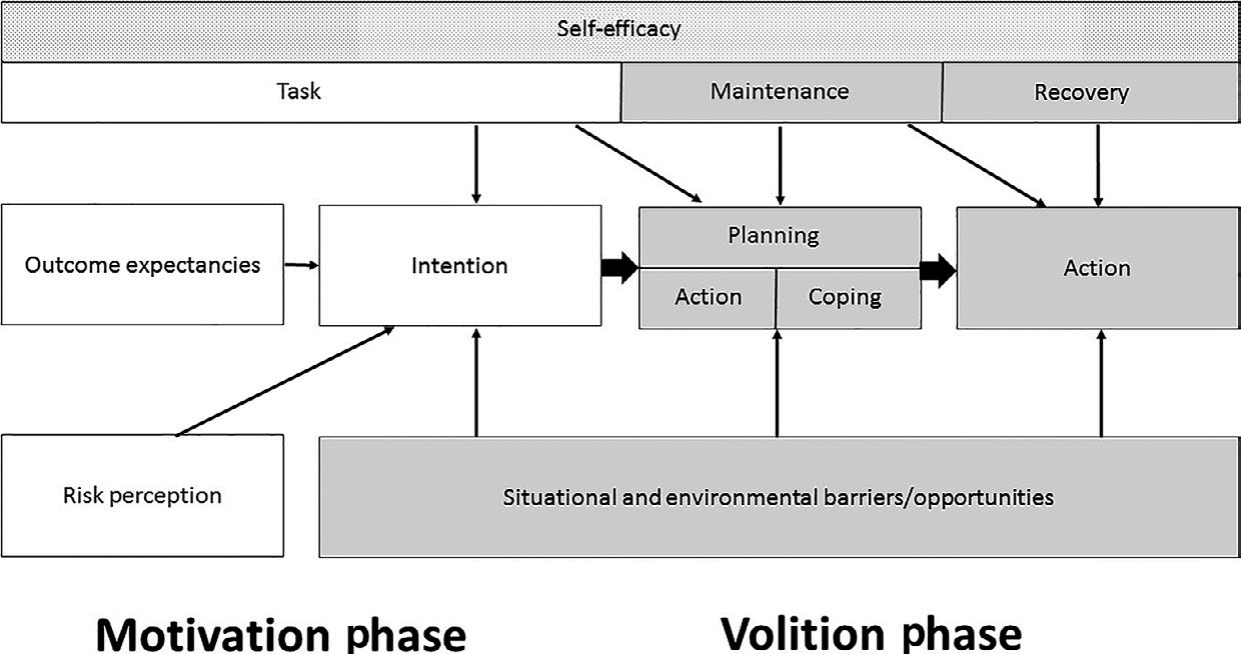
Health Action Process Approach (HAPA) model^21^

Given the constraints of the study and relatively low number of participants, a coding strategy using emerging data saturation analysis was felt unlikely to be achievable and therefore elements of the HAPA model were used directly as a framework to investigate oral hygiene behaviour in diabetes patients^28^, ^29^.The HAPA model comprises two consecutive behavioural phases. The motivation phase in which the psychological constructs “risk perception,” “outcome expectations” and the actual “task” of the behaviour all act on the “intention” of behaviour change. This is followed by the volition phase in which the “intention” is transformed into “action.” Both phases are dependent on the overarching construct, “self‐efficacy” (Figure 1). Risk perception alone is not sufficient for a person to form an intention but starts a process of deliberating consequences and competencies. Outcome expectancies are a consideration of the pro and cons of certain behavioural outcomes. Together with the perceived self‐efficacy, both are essential for forming the intention of behavioural change and to enter the volitional phase. Once an intention is formed, this has to be transformed into detailed instructions how the action will be carried out, involving self‐regulatory skills and strategies.^23^

The interviews were coded using elements of the HAPA model constructs as key themes instead of developing themes from the data^28^, ^29^. In the following step, examples for these themes were marked together electronically using NVivo.

Initial mapping of the data against the HAPA constructs was discussed amongst the co‐authors for cross‐checking of data interpretation and refinement of the analysis. Specifically, the somewhat uncommon approach of not coding for emerging themes was discussed and weaknesses such as the potential of missing out on some important information were acknowledged, bearing in mind that the present study is a feasibility study.

### 2.5 Statistical analysis

This work is a feasibility study and was not informed by a power calculation. Therefore, a formal statistical analysis was not conducted.

### 2.6 Ethical approval and sources of funding

Informed written consent was collected from all participants of this study and the study was conducted in accordance with the Declaration of Helsinki.^30^ The study was given a favourable ethical opinion by the Liverpool Central Research Ethics Committee (ref 15/NW/0294).

The study was funded from a UK National Institute of Health Research (NIHR) Transitional Research Fellowship (TRF‐2014‐07‐003).

## 3 RESULTS

### 3.1 Clinical data

Table 1 shows general and clinical characteristics of the study population. The data suggest improvement, albeit moderate, in the periodontal condition 3 months post‐treatment compared to pre‐treatment. Reductions were noted in median PD and CAL, %BOP, the median number of sites with PD ≥ 5 mm, and median PESA and PISA scores.

### 3.2 Qualitative analysis: HAPA model application

**Figure 2:**
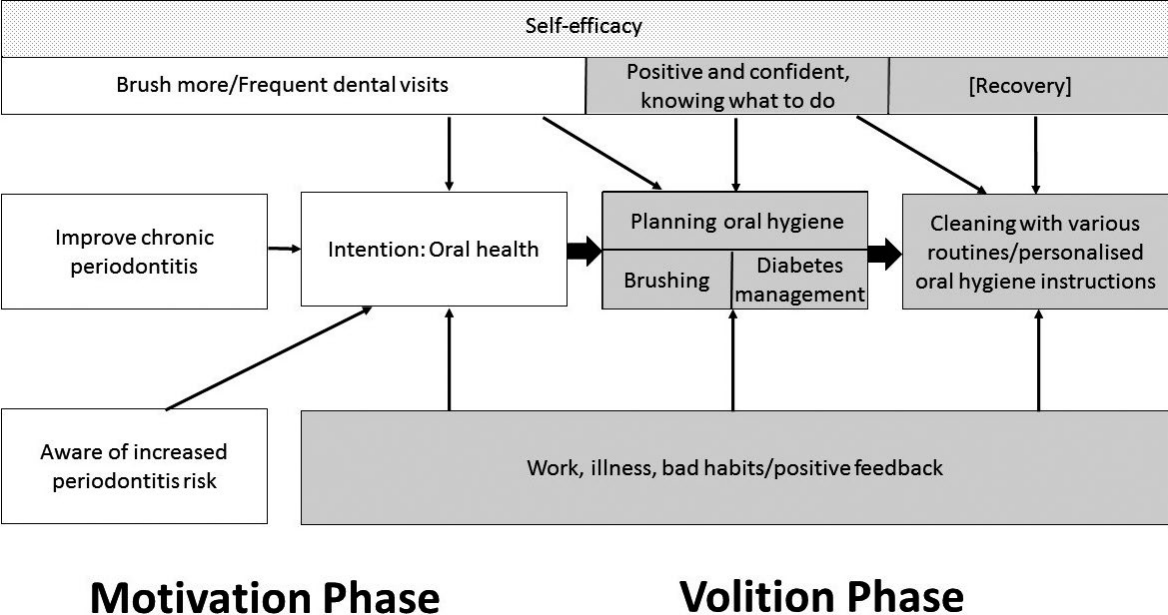
Application of the HAPA model to the behavioural change pathway of periodontitis patients with diabetes

Applying the HAPA model to the behavioural change pathway in patients with periodontitis and diabetes can be summarized as shown in Figure 2. Specifically, patients moved through the following phases before, during and after periodontal treatment:

### 3.3 Motivation phase

#### 3.3.1 Intention

The key intention of patients participating in the study was to achieve a consistently high level of good oral health and long‐term control of their periodontitis. This is reflected in data illustrating their outcome expectancies, risk perception and task self‐efficacy, which come together to act on the intention of improving their oral hygiene.

#### 3.3.2 Outcome expectancies

Participants were actively seeking a referral to Newcastle Dental Hospital with the view to improve their periodontitis and to overcome concerns:

“I was not happy about the number of teeth which I was losing, my own dentist, he didn’t seem to care whether I was losing teeth or not. So I asked for a referral.” (M3)

“Basically ‘cos I pushed my dentist to make a referral because my gums weren’t getting any better I felt, for my age, I had concerns (…) of nothing was, looked into then it would, progress.” (F6)

“The infections in me gums. Um, I was um, my dentist discovered it was leaking pus. Um, infections so they’ve, forwarded me to the dental hospital (…) for, like, x‐rays and, a consultant, yeah, opinion.” (M7)

These outcome expectancies demonstrate the patients’ willingness to act on their poor oral health by taking the initiative of asking their dentist for a referral to the dental hospital.

#### 3.3.3 Risk perception

Suffering from diabetes, participants commonly knew of their increased risk of developing periodontitis, made aware by their dentist or through self‐education:

“From my previous dentist, he didn’t mention anything about diabetes and, gum disease but, the new dentist that I started with, she pointed it all out to me.” (M7)

“I knew, because I am also a doctor myself, so I ... I was aware. Yeah, I was aware.” (M4)

“I could tell, when I looked in the mirror, at my teeth that something wasn’t right, but I didn’t know what it was. I thought it was, part of getting old, receding gums, um, it was normal, it was something that happens to people when they get older. But I’ve since been told from my dentist, that’s not the case.” (M1)

The perception that diabetes patients had about their risk of developing periodontitis is setting the scene to making changes in their oral self‐care.

#### 3.3.4 Task self‐efficacy

Study participants were referred to the Newcastle Dental Hospital with a diagnosis of recurrent or difficult to manage periodontitis. Frequent dental visits were observed regularly:

“Every half year, but I’ve been told I should really be going back every three months.” (M3)

“Oh, every six months say, roughly, twice a year. Yeah.” (F3)

Equally, frequent tooth brushing routines were already widespread in this patient group:

“Yeah. Yeah. Twice a day. Yeah. On a morning, and before bed.” (F1)

“(…) first, after breakfast and then last thing at night.” (M2)

“Yes, well I’ve always cleaned my teeth, every morning and every evening before going to bed. Every morning before breakfast, every evening before going to bed.” (M5)

These quotes show the perceived self‐efficacy of patients to do their best with oral self‐care through regular dental visits and regular tooth brushing.

### 3.4 Volition Phase

#### 3.4.1 Planning: action and coping

Participants indicated that they did not find it difficult to plan their new personalized oral hygiene regime around their diabetes management:

“It’s had no impact at all. I mean, it takes a fraction longer but it’s not impacted at all.” (M2)

“I get up in the morning, I go in the bathroom, and, check my sugar level. Then I normally have an injection, and, go in the shower, have breakfast, go back in (…) I come back upstairs, clean my teeth, then I go away for the day.” (M3)

Rarely did participants prioritize one over the other:

“Um, well, when I was working, not working at the moment, but I’d made sure I’d get up in plenty of time to, have my breakfast, do my blood sugars, and then do my teeth after that. Before I set off for work. (…) ‘Cos I make sure like my diabetes‐ diabetes is controlled first. Um, I know the teeth is important as well, but my blood sugars are important.” (F1)

The postintentional factors of planning for the action and coping show that patients had the right skills to implement the action of oral self‐care within their diabetes management.

#### 3.4.2 Action

Commonly, participants were able to give a detailed account of their oral hygiene routine:

“(…) most important brushing is in the night time, where I brush my teeth, using the method of, I don’t know how you describe it (…), brushing away from the gums, not brushing up and down (…) so I take any infection out, out the gum area, um, top and bottom, using the TePe’s to get into the gaps as best I can (…). I’m using at the moment, yellow, purple and green. That’s me three main colours, for getting into the gaps. I have got some big gaps, in my gums, where me gums have receded but I find the purple and green get right in.” (M1)

“I use the electric toothbrush, and then I go along the gum lines with a manual [toothbrush], and then I use the interdental brushes, and then I floss where I can’t get the interdental brush twice a day.” (F6)

Study participants frequently reported a change in their tooth cleaning regime after receiving the personalized oral hygiene instructions as part of their periodontal treatment:

“Like I was using mouthwash after I brushed but, um, like I say I was told not to use it anymore because it brush‐, it’s washing away all the stuff I’ve, I’m doing, so.” (F1)

“I didn’t used to use the brushes. So that’s been added.” (M3)

“Definitely, I have adopted your measures, um, only that probably I’ve not been as effective as I should have been, especially, the front gums whereby, I think I’m not really getting, been getting under, the... because I’ve, I’ve been told I’ve got to use pressure, when I apply the brush, close to the gums so that I brush, with pressure. I was not using pressure. I was just going up and down.” (M4)

“Um, the only thing I’ve added was the um, like the single, bristle brush, yeah.” (M7)

The action of implementing the OHI is continuously controlled through self‐regulating behaviour by comparison of what patients have been doing in the past and what they are doing now.

#### 3.4.3 Maintenance self‐efficacy

Incorporating the personalized oral hygiene techniques into a daily regime was a recurrent finding amongst study participants and they appeared positive and confident:

“Because I think I’ve got my routine. [laughs] You know, absolutely spot on, I wake up in the morning, depending on what day it is, and thankfully, I don’t have to, have my, you know, my breakfast at a certain time etc., I eat when I want to eat, and whenever I have something to eat I brush my teeth and then I do exercises (…)” (F3)

“I’m, I’m used to having to fit stuff in so, [laughs] with my diabetes and things, and I just, it’s something that’s important to me, that I want to make sure my health is controlled including my teeth, so it’s, I didn’t really think about it.” (F6)

“Well, um, when I get up in the morning it’s the first thing that I do, (…) and it’s, like, last thing I do before I go to bed. I just like, do it on autopilot now.” (M7)

The quotes demonstrate that patients reach self‐efficacy in maintaining the OHI to the point of feeling confident about their ability.

#### 3.4.4 Situational and environmental barriers/opportunities

Study participants predominantly described bad habits and other illnesses as causes for missing their usual oral hygiene routine:

“I always think I could do better, um, sometimes um, if not in a rush but, I’ll always do the electric tooth brushing, but not always the bottle brushing in the morning. I try to, but sometimes, think I’ll have that extra five minutes in bed… it just takes, you know, that’s… that’s probably the only time it suffers.” (M2)

“(…), just like, last week, and um, the week before when I caught that winter vomiting, bug, and um, then the head cold afterwards. Um, sort of like, it made me extremely lethargic, I couldn’t be bothered with cleaning me teeth, I didn’t cook much in the way of food, ‘cos it’s just like, I just like, lay on the couch all day or me bed, um, feeling sorry for myself.” (M7)

Whereas work did not appear to be an important barrier:

“No, there, um, the shift, the shift patterns I work, aren’t, don’t um, affect, any regime in brush, brushing my teeth. I can still carry on the normal brush, um, brushing regime.” (M1)

Positive feedback from their treating clinician on how well study participants were doing with following their personal oral hygiene was rarely mentioned as a contributor for maintaining the personalized oral hygiene regime:

“Um, but it’s nice to make‐ make sure I’m doing it right.” (F1)

These patients’ quotes recognize how situational and environmental barriers and opportunities can impact on aspects of planning and action control.

#### 3.4.5 Recovery self‐efficacy

Study participants did not report to be moving through a “recovery” phase as this was not applicable within the short time‐frame of the study.

## 4 DISCUSSION

It is well recognized that adherence to behaviours that optimize oral hygiene are an essential for achieving successful outcomes for periodontal disease treatment.^2^, ^3^ Complex psychological interventions (such as group sessions, reinforcement, goal setting, self‐monitoring, provision of feedback and motivational interviewing) conducted by psychologists or specifically trained dental personnel have been trialled, with limited success in inducing a behavioural change in oral hygiene.^31^, ^32^ Notably, meta‐analyses evaluating how such a change in behaviour is best brought about criticize the weak evidence‐base.^6^, ^7^ Clinical data indicate a positive treatment outcome in this study, albeit only monitored for a short time period. This was achieved following a standard personalized approach for improving oral hygiene, including “every day,” oral hygiene advice as is routinely undertaken in primary care dental practice. This is consistent with the general practice of a 3‐monthly recall for periodontitis patients during the maintenance phase of care.^4^ It is, however, questionable if this approach would change oral hygiene behaviours in the longer term, yet, to date, evidence for this remains weak even when utilizing complex psychological interventions.^6^, ^7^, ^31^, ^32^

Importantly, the present study was able to demonstrate that it is feasible to map interview data on oral hygiene behaviours with psychological constructs of the HAPA model. This is an important finding as it allows for a novel type of data analysis which may be applicable to other scenarios where a behavioural change is intended, such as for example dietary or exercise interventions or adherence to medication regimes. This methodology may also be applied to other behavioural change models and not just the HAPA model.

The present approach is in contrast to previous studies on this topic in which only quantitative data such as clinical indices or questionnaires were collected,^18^, ^20^, ^22^ thereby not considerating patients’ feelings and emotions and potentially not identifying how patients struggle to adhere to a behavioural change. Specifically, our results show that patients’ concerns about losing teeth, their knowledge of increased risk for severe periodontitis and frequent dental visits and tooth brushing all contributed to the intention to improve their oral hygiene behaviour. Despite having to consider their diabetes management at the same time, patients were able to implement new oral hygiene procedures without great difficulty and were able to maintain these throughout the trial period by incorporating them into their daily routine (“routinisation”) and by maintaining self‐confidence. Possibly, this patient group had already experienced substantial behaviour changes when learning how to manage their diabetes which may have helped them to incorporate a new behavioural routine into their daily lives. It is interesting to note that occasionally, patients described a hierarchy of prioritizing their diabetes care over their oral hygiene care if there was a conflict how to manage both simultaneously. At other times, seemingly trivial “bad habits” such as staying in bed 5 minutes longer appeared to have a recognizable effect on their oral hygiene management whilst a considerably more extensive daily interruption, such as shift work, was of little concern. A future study should explore these issues in more detail with the aim to achieve full data saturation in patient interviews.

To the best of our knowledge, the present study evaluates, for the first time, oral hygiene behaviour change specifically in patients with periodontitis and diabetes. Diabetes management itself requires several adjustments by patients such as changes in diet, exercise, frequent checks of blood glucose levels, and routine medical appointments.^10^ The expectancy, therefore, was that patients may struggle with the additional task of improving their oral self‐care. However, surprisingly, none of the patients reported this to be a point of concern to them. On the contrary, patients stated that due to their complex diabetes management, they were already used to “fitting stuff in” and therefore implementing the new oral hygiene procedures had little impact on their daily routine.

The current study was conducted as a feasibility study and is therefore limited in the conclusions that can be drawn. Notably, the study group was small and future work should consider broadening the recruitment range to take account of influences of socioeconomic and demographic factors, including smoking and body weight, on any behavioural change the patients may undertake. Taking into consideration that, often, the behavioural change is short‐lived,^33^ a future study should have a longer follow‐up period. Also, some patients saw the hygienist twice for their treatment, others only once which potentially may have introduced some form of bias in re‐enforcing OHI. Furthermore, patients were recruited from periodontology referral clinics. This may infer that they already had a higher intention for a behavioural change in their OHI than the average dental patient. In addition, it would be interesting to repeat interviews over a longer time period to identify whether adherence to the new behaviours changes over time. Future work should also consider a dual approach of applying the HAPA model to both quantitative and qualitative data to identify any results not covered by interview analysis and vice versa.

In summary, through application of the HAPA model to interview data, this study evaluated a novel approach to explore changes in oral hygiene behaviour in patients with diabetes and periodontal disease. Specifically, this approach allowed for the collection of richer data and these preliminary findings suggest that in general, patients with periodontitis and diabetes successfully manage to incorporate new oral hygiene behaviours into their daily routine. It remains to be elucidated if these behavioural changes persist in the longer term and if some of the barriers revealed by the interview data analysis would impact on this.

## 5 CLINICAL RELEVANCE

### 5.1 Scientific rationale for study

Patients with diabetes undergoing periodontitis treatment have to manage both their oral hygiene and diabetes control. It is not known if there are barriers preventing these patients from implementing a successful oral hygiene routine.

### 5.2 Principal findings

A routine approach of non‐surgical periodontal therapy and personalized oral hygiene instruction (an overview of periodontal risk‐factors, tooth brushing instructions and provision of interdental brushes, tailored to each patient's clinical needs) delivered by a dental hygienist are likely sufficient for patients with periodontitis and diabetes to successfully incorporate new oral hygiene behaviours into their daily routine.

### 5.3 Practical implications

Research into improving oral hygiene behaviour should focus on everyday routine standard dental practice and incorporate more qualitative data to achieve a richer data outcome.
